# Facial affect and arousal as a complement to gaze measures in infant speech sound perception studies

**DOI:** 10.3389/fncir.2025.1553918

**Published:** 2025-05-30

**Authors:** Sho Tsuji, Fernanda Alonso, Hiromichi Hagihara, Nanako Kimura, Linda Polka, Irena Lovčević

**Affiliations:** ^1^Laboratoire de Sciences Cognitives et Psycholinguistique, ENS, EHESS, CNRS, PSL University, Paris, France; ^2^International Research Center for Neurointelligence (WPI-IRCN), The University of Tokyo, Tokyo, Japan; ^3^School of Communication Sciences and Disorders, Centre for Research on Brain, Language and Music, McGill University, Montreal, QC, Canada; ^4^Graduate School of Human Sciences, The University of Osaka, Suita, Japan; ^5^Graduate School of Engineering, The University of Tokyo, Tokyo, Japan

**Keywords:** speech perception, infancy, perceptual attunement, facial affect, automatic facial analysis

## Abstract

This study explores infant facial expressions during visual habituation to investigate perceptual attunement to native and non-native speech sounds. Using automated facial affect analysis based on Facial Action Units, we analyzed valence, arousal, positive affect, and negative affect during the experiment. Valence and arousal decreased with habituation, while positive affect increased, with differences between native and non-native stimuli. Facial affect showed links to discrimination outcomes, with better native discrimination linked to reduced negative affect. These findings highlight the potential of facial expression analysis as a complementary tool to gaze-based measures in early language development research.

## 1 Introduction

Human infants’ ability to learn the phonemes of their native language relies on neural mechanisms of perceptual attunement. Initially capable of discriminating a wide range of phoneme contrasts across world languages, infants typically show a decline in sensitivity to non-native phonemes and an improvement in native phoneme discrimination within the first year of life (Werker and Tees, 1984). This experience-dependent tuning reflects synaptic pruning and neural plasticity during critical developmental periods and is positively linked to later language skills (Werker and Hensch, 2015).

To study perceptual attunement, infants are typically habituated to a specific speech sound, and dishabituation to a novel sound is measured via looking times. This method robustly demonstrates perceptual attunement across languages and cultures (Tsuji and Cristia, 2014).

However, perceptual attunement is not a uniform process, and factors such as the richness of the linguistic and social environment shape its trajectory. For instance, bilingual infants may take longer to attune their speech sound categories compared to monolingual infants (Bosch and Sebastián-Gallés, 2003), and socially enriched learning environments enhance infants’ acquisition of speech sound contrasts (Kuhl et al., 2003). These findings suggest that attunement is influenced by the interplay of linguistic exposure, social interaction, and cognitive development.

Given these multifaceted influences, it could be insightful for discrimination measures to also capture the richness of infants’ responses. While the conventional looking time measure is invaluable, it may miss other signals, such as facial expressions, that provide additional insight into the affective and cognitive processes underlying attunement. Video recordings of infants’ faces in habituation studies offer untapped potential for such analyses.

Advances in automated analysis tools, such as FaceReader and its infant-specific adaptation BabyFaceReader (Noldus Information Technology, 2021), enable the systematic extraction of facial expressions using the Facial Action Coding System (Ekman and Friesen, 1978). This framework quantifies affective states by identifying discrete facial muscle movements, or action units (AUs). Affective expressions can be classified within a bidimensional framework of valence and arousal: valence reflects the intrinsic positivity or negativity of a state, while arousal represents its intensity or activation level. During habituation, arousal is expected to decrease and then increase in response to a novel stimulus (e.g., Thompson, 2009), while valence may vary depending on factors such as age and stimulus complexity (e.g., Hunter and Ames, 1988). In addition to valence and arousal, specific AU configurations allow the assessment of negative affect (unpleasant emotions) and positive affect (pleasant emotional states), which have been found to be closer aligned to manual coding of affect (Zaharieva et al., 2024) and which can provide a more granular view of infants’ affective state.

By analyzing these dimensions, we aim to enrich our understanding of the affective and attentional responses associated with perceptual attunement. Our study focuses on Japanese infants’ developing discrimination of two phonemic contrasts: a native vowel length contrast and a non-native r-l contrast. Both have been documented to exhibit characteristic patterns of perceptual attunement, with studies using the same native contrast and testing method showing improved discrimination between 6 and 12 months. Six-month-olds failed to discriminate the contrast, while 12-month-olds succeeded (Lovčević and Tsuji, 2024; Sato et al., 2010). Results for 9-month-olds were mixed, with successful discrimination reported by Lovčević and Tsuji (2024) but not Sato et al. (2010). For the non-native r-l contrast, studies have consistently shown a decline in discrimination over the first year. Japanese infants aged 6–8 months could discriminate the contrast, but this ability diminished by 10–12 months (Kuhl et al., 2006; Tsushima et al., 1994). Using the same contrast and method as in the present study, Lovčević and Tsuji (2024) reported successful discrimination at 9 months but not at 12 months.

The current dataset includes longitudinal video data on infants’ discrimination of the native and non-native contrast at 9 and 12 months. By analyzing facial expressions during speech sound discrimination, we aim to reveal the potential of integrating this measure into the study of perceptual attunement.

## 2 Methods

### 2.1 Participants

All experiments were conducted within-subjects, but not all combinations of infant and data points could be retained. For looking time data in the native speech sound discrimination experiment, data from 31 9-month-old infants (range 246–297 days, 16 female) and 33 12-month-old infants (range 367–395 days, 18 female) were included. A further five 9-month-old infants and one 12-month-old infant were excluded from analysis due to not completing the task. For the non-native speech sound discrimination experiment, data from 29 9-month-old infants (range 246–297 days, 14 female), and 29 12-month-old infants (range 365–395 days, 15 female) were included. Six 9-month-old and two 12-month-old infants were excluded from analysis due to not completing the task or not habituating.

For the facial expression analyses, we excluded participants who were missing video recordings due to experimenter or equipment error or missing parental approval. The sample for native speech perception included 20 9-month-old infants (range 246–293, 14 female) and 24 12-month-old infants (range 367–395, 12 female). As for non-native speech perception, the final sample included 18 9-month-old infants (range 246–293, 12 female), and 22 12-month-old infants (range 365–395, 11 female).

All infants were monolingual Japanese recruited in Tokyo, and caregivers gave written informed consent for participation and data reuse before the experiment. The protocol was approved by the local IRB committee (name anonymized for submission).

### 2.2 Stimuli

Stimuli consisted of native and non-native speech sounds, and were identical to those in Lovčević and Tsuji (2024). The native contrast consisted of non-words /mana/ and /ma:na/, differing in vowel duration. The /ma:na/ stimuli were recorded by a female Japanese speaker, while /mana/ stimuli were generated by shortening the steady part of /a:/ to control acoustic differences. The non-native contrast used the English words “right” (/ɹaɪt/) and “light” (/laɪt/), unfamiliar to Japanese infants, and recorded by a female Canadian English speaker. Stimuli were recorded in infant-directed register and presented in lists of 10 repetitions with ~1.5-s intervals. Since this study involved affect-related facial expressions, six adults rated the valence of the stimuli (following Kitamura and Burnham, 2003, but without low-pass filtering). While adult ratings may not fully capture infant perception – particularly as non-native stimuli consist of real words, potentially influencing judgments – they provide some insight into valence differences. Median valence was higher for native (1, IQR = 3) than non-native stimuli (0, IQR = 1.25), with ordinal regression confirming this difference as significant (Odds ratio = 0.452, *p* = 0.035). Since native stimuli were disyllabic and thereby longer than non-native monosyllables, they might have provided more possibility to express affect.

### 2.3 Procedure

Each experimental session consisted of one experiment using native, and one experiment using non-native stimuli. Each infant participated in two sessions (at 9 and 12 months). Experiments took place in a dimly lit, soundproof lab and were controlled using Habit X 2.0 software (Oakes et al., 2019) on a Windows computer. Visual stimuli were displayed on a 23-inch screen, and audio stimuli were delivered via forward-facing speakers below the screen. Infants sat on their caregiver’s lap, while caregivers wore noise-canceling headphones playing music to mask the auditory stimuli. An experimenter observed the infant through a video camera and recorded their gaze to the screen by pressing a key on the computer keyboard, with key presses logged for analysis. Infants’ gaze was also recorded on video.

The study used a modified visual habituation paradigm (Stager and Werker, 1997) with four phases: pre-test, habituation, test, and post-test. Before each trial, an attention getter was presented, and as soon as the infant looked to the screen, the experimenter pressed the key on the experimental computer keyboard to start the next trial. Pre- and post-test consisted of one trial each, featuring a rotating wheel animation paired with the audio stimulus “panta.” Comparing looking behavior in these phases assessed potential fatigue or disinterest.

Presentation order of the native and non-native experiment was counterbalanced across infants, and infants took a short break between experiments. Within the native or non-native experiment, half of the infants were habituated to one type of speech sound (/mana/ or /ma:na/ for the native, and /ɹaɪt/ or /laɪt/ for the non-native experiment).

In each experiment, during habituation, stimulus lists representing one type of speech sound were presented accompanied by a static red-and-black checkerboard visual. Habituation ended when infants met the criterion (average looking time in a four-trial sliding block <60% of the first block) or completed 28 trials.

In the test phase, infants completed four trials: two “switch” trials with the respective other speech sound, followed by two “same” trials matching the habituation stimulus. For example, infants habituated to /ma:na/ heard /mana/ in switch trials and /ma:na/ in same trials.

### 2.4 Data preprocessing

To analyze infant gaze behavior, we extracted logged keypress data, indicating looks to and away from the screen during each trial.

Facial expressions were analyzed using BabyFaceReader (Noldus Information Technology, 2021), which detects infant faces, maps facial landmarks with a mesh, and analyzes gaze, head orientation, and facial movements frame-by-frame. BabyFaceReader then generates outputs for all AUs and combines these to code specific expressions, in our case valence and arousal. Valence, ranging from −1 (maximum negative emotion) to +1 (maximum positive emotion), reflects overall emotional tone, while arousal, ranging from 0 (minimum) to 1 (maximum), indicates intensity. In addition to these valence scores (which combine many AUs), we analyzed positive and negative affect using selected AUs. Negative affect, derived from AU3 and AU4 (brow lowering), and positive affect, represented by AU12 (lip corner puller), range from 0 (low) to 1 (high) (Maroulis, 2018; Noldus Information Technology, 2021), and were added to the analyses for two reasons. First, a recent study by Zaharieva et al. (2024) found a closer alignment between manual and automated coding of positive affect when using AU12 compared to using the composite valence score. Similarly, they also found good performance when using AU3 + AU4 to categorize negative affect. Second, there are open-source tools that measure these specific action units, whereas BabyFaceReader is costly and hence, not widely available.

The videos were recorded continuously during each experiment and were not synced with the experimental software, and therefore did not include trial start and end times. To address this, human annotators manually coded the onset of experimental phases (pretest, habituation, test, and post-test) in the videos based on sound onsets. This timing information was then integrated with trial onset data from the experimental software and aligned with the BabyFaceReader output.

### 2.5 Analysis plan

We first analyzed gaze behavior to assess perceptual attunement, following Lovčević and Tsuji (2024). Linear mixed-effects models were fitted separately for the native and non-native experiments in each age group using the *lmer* function from the lme4 package (Bates et al., 2015) in R (R Core Team, 2013). The models included Trial (same, switch), Habituation Stimulus (native: /mana/, /ma:na/, non-native: /ɹaɪt/,/laɪt/), and their interaction as fixed effects, with random intercepts for participants and infants’ looking time as the dependent variable. Significance was tested using ANOVAs with Satterthwaite’s method via the anova function of the *lmerTest* package (Kuznetsova et al., 2017).

We next conducted three sets of confirmatory analyses on facial expressions, each comprising four linear mixed effects models with valence, arousal, negative affect, or positive affect as the dependent variable. Categorical predictors common to all analyses were Age (9, 12 months) and Nativeness (native, non-native), and were sum-coded. Models included random intercepts for participants. Significance was tested with Type III ANOVAs (*car* package, Fox and Weisberg, 2019), and *post hoc* pairwise Bonferroni-corrected comparisons were performed (*emmeans,* Lenth, 2019). Visualizations used *ggplot2* (Wickham et al., 2016).

First, to track changes in facial expressions during the habituation phase, we assessed the effect of Age, Nativeness, Trial Number, and their interactions. The continuous predictor Trial Number (1–8) reflected the habituation trial number, where, to avoid sparse data, we excluded trials beyond the point where trial counts dropped below 50% of the initial count (trial 8). This predictor was centered.

Second, we examined differences in facial expressions between same and switch trials in the test phase, focusing on subsets likely to show behavioral discrimination. A group-based subset included 12-month-olds exposed to native and 9-month-olds exposed to non-native contrasts, as these groups are expected to show discrimination (Lovčević and Tsuji, 2024). An individual-based subset included all infants with an above-chance Novelty Score, a score reflecting the degree to which infants look more to trials with the novel compared to the known (habituated to) stimulus. The Novelty Score is calculated as looking time to switch trials divided by total looking time during switch and same trials [as in Arterberry and Bornstein (2002)]. Models included Age, Nativeness, and Phase (same, switch).

Third, to assess whether facial expressions showed any relationship to infants’ discrimination performance, we conducted an exploratory analysis predicting facial expressions during habituation based on infants’ Novelty Score. Facial expression values during habituation were modeled as a function of Novelty Score and Age.

## 3 Results

### 3.1 Speech perception

#### 3.1.1 Native speech perception

Infants both at 9 and 12 months demonstrated significant discrimination [9 m: *F* (1, 87) = 5.03, *p* < 0.05; switch: *M* = 6,655 ms, SD = 2,952, same: *M* = 5,288 ms, SD = 2,993; 12 m: *F* (1, 82) = 5.17, *p* < 0.05; switch: *M* = 5,926 ms, SD = 2,897, same: *M* = 4,709 ms, SD = 2052]. We note that another group of 6-month-olds (not included in this manuscript) did not show evidence of successful discrimination; thus, these findings align with patterns found in the perceptual attunement literature.

#### 3.1.2 Non-native speech perception

Infants at both 9 and 12 months showed no significant discrimination [9 m: *F* (1, 84) = 3.45, *p* = 0.07; switch: *M* = 4,946 ms, SD = 3,460, same: *M* = 4,078 ms, SD = 1966; 12 m: *F* (1, 85) = 0.40, *p* = 0.53; switch: *M* = 3,892 ms, SD = 2071, same: *M* = 4,197 ms, SD = 2,474]. Based on previous work using the same stimuli (Lovčević and Tsuji, 2024) we would have expected discrimination at 9 months; however, the *p*-value suggests a tendency in a direction consistent with perceptual attunement.

### 3.2 Facial expressions

#### 3.2.1 Habituation phase

**Figure 1:**
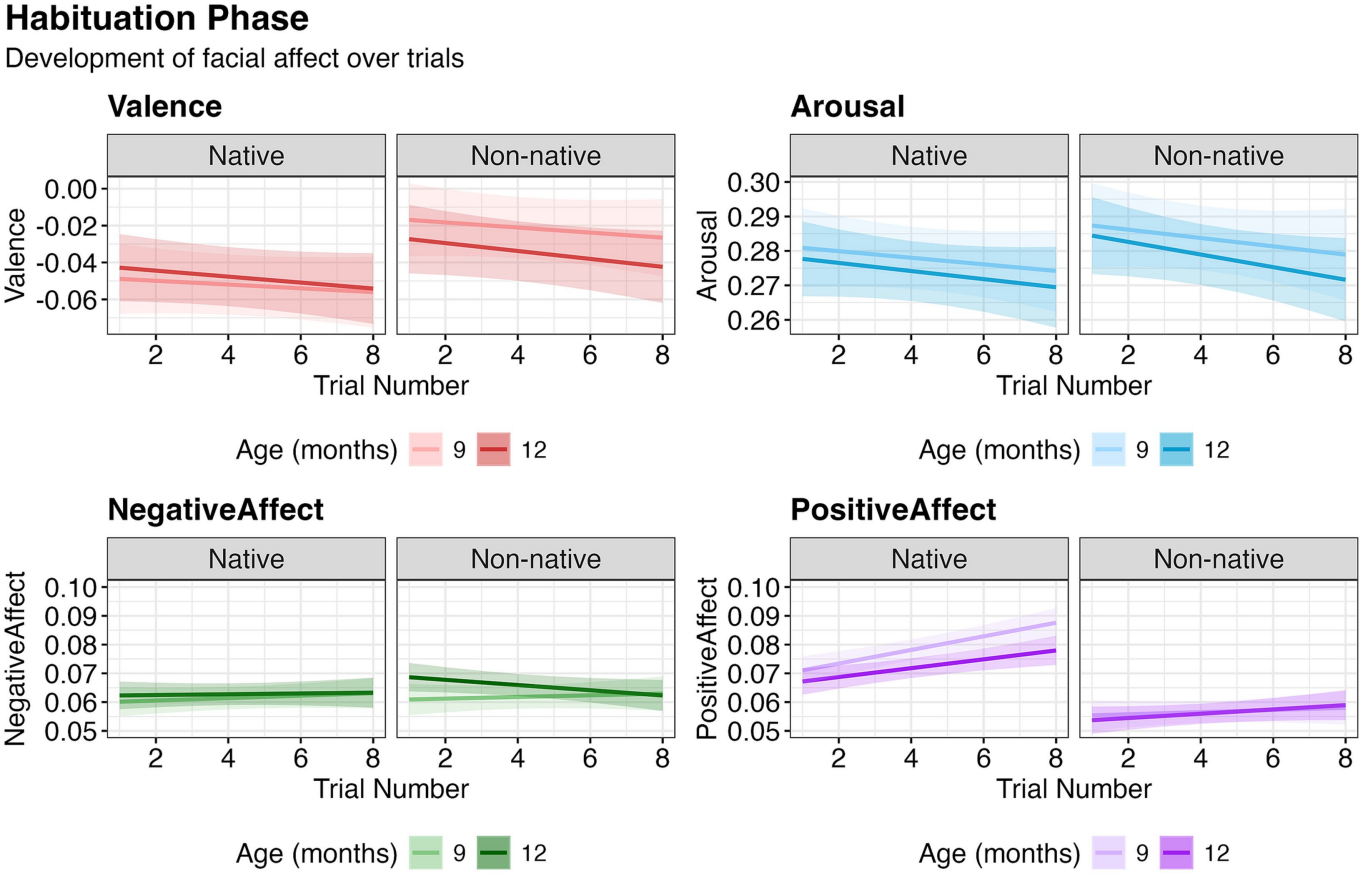
Development of facial affect over the course of habituation trials. Relationship between affect and trial number based on model estimates. The shaded region represents the 95% confidence interval around the fitted values.

Valence decreased significantly with increasing Trial Number [*χ*^2^(1) = 4.19, *p* = 0.043]. Nativeness had a significant effect [*χ*^2^(1) = 43.31, *p* < 0.001], with valence higher in response to non-native (*M* = −0.030, SD = 0.039) compared to native (*M* = −0.050, SD = 0.069) stimuli. Age interacted with Nativeness [*χ*^2^(1) = 6.31, *p* = 0.012], and post-hoc paired comparisons showed that the Age difference was significant for non-native [*t* (590) = 2.36, *p* = 0.019], but not for native stimuli [*t* (590) = −0.82, *p* = 0.414]. Arousal attenuated with increasing Trial Number [*χ*^2^(1) = 5.28, *p* = 0.022], and was higher for non-native (*M* = 0.280, SD = 0.030) than native (M = 0.276, SD = 0.040) stimuli [*χ*^2^(1) = 4.16, *p* = 0.041]. Negative affect did not change over trials [*χ*^2^(1) = 0.01, *p* = 0.091]. Positive affect increased with increasing Trial Number [*χ*^2^(1) = 22.92, *p* < 0.001]. Age had a significant effect [*χ*^2^(1) = 6.65, *p* = 0.010], with higher positive affect at 9 months (*M* = 0.069, SD = 0.021) than at 12 months (*M* = 0.066, SD = 0.016). Positive affect was higher for native (*M* = 0.076, SD = 0.022) than for non-native (*M* = 0.057, SD = 0.006) stimuli [χ^2^(1) = 262.51, *p* < 0.001]. Trial number interacted with nativeness (χ^2^(1) = 6.97, *p* = 0.008), but post-hoc analyses showed that positive affect increased significantly with increasing trial number for both native [*χ*^2^(1) = 22.51, *p* < 0.001] and non-native [*χ*^2^(1) = 17.29, *p* < 0.001] stimuli separately. Age interacted with nativeness [χ^2^(1) = 6.62, *p* = 0.010], and follow-up paired comparisons revealed that the effect of age was only significant for native [*t* (595) = 3.76, *p* < 0.001], but not non-native stimuli [*t* (595) = 0.25, *p* = 0.803] (Figure 1). No other effects were significant. These findings suggest that facial affect reflects the habituation process, including decreases in valence and arousal and increases in positive affect, with variations across native/non-native stimuli and infant age.

#### 3.2.2 Test phase

No significant differences were found between same and switch trials across facial expressions in the group-based analysis [valence: χ^2^(1) = 0.85, *p* = 0.356; arousal: χ^2^(1) = 0.91, *p* = 0.338; negative affect: χ^2^(1) = 0.08, *p* = 0.784; positive affect: χ^2^(1) = 0.05, *p* = 0.822]. Valence was significantly influenced by group (χ^2^(1) = 7.38, *p* = 0.007), with 9-month-olds exposed to non-native stimuli (*M* = −0.031, SD = 0.046) showing higher valence than 12-month-olds exposed to native stimuli (*M* = =0.047, SD = 0.061). Positive affect also was significantly influenced by group [*χ*^2^(1) = 11.75, *p* < 0.001], with 12-month-olds exposed to native stimuli (*M* = 0.076, SD = 0.020) showing higher positive affect than 9-month-olds exposed to non-native stimuli (*M* = 0.068, SD = 0.031).

**Figure 2:**
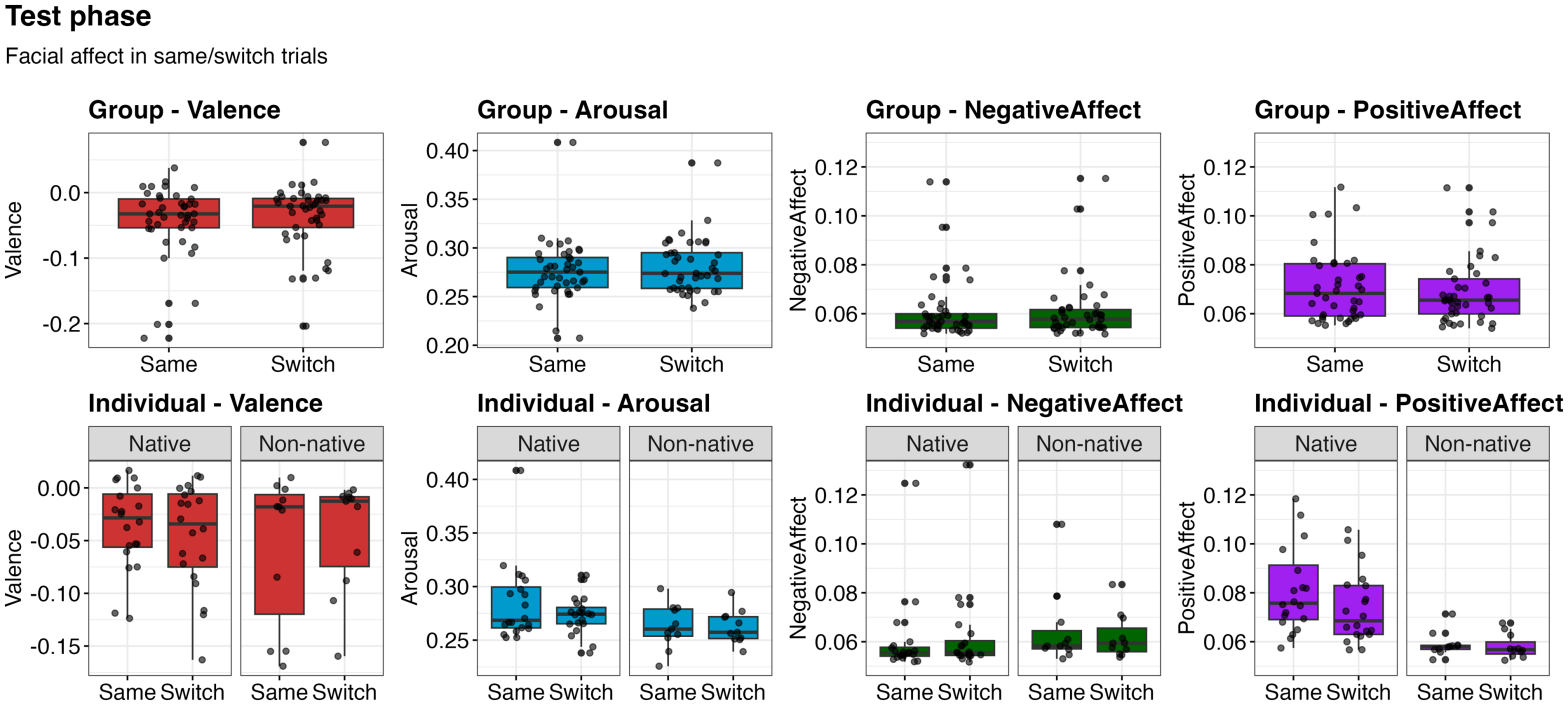
Facial affect values during the same and switch trials of the test phase. Upper row represents analyses on group-based subset, and lower row represents analyses on individual-based subset. Boxes represent the interquartile range (IQR), with the median indicated by a horizontal line. Whiskers extend to 1.5 times the IQR, and black points beyond the whiskers represent outliers. Grey shaded data points represent participant means and are overlaid with jitter for visualization of individual observations.

Similarly, no significant differences between same and switch trials were found in the individual-based analysis [valence: *χ*^2^(1) = 0.05, *p* = 0.823; arousal: *χ*^2^(1) = 0.98, *p* = 0.321; negative affect: *χ*^2^(1) = 0.140, *p* = 0.708; positive affect: *χ*^2^(1) = 1.72, *p* = 0.189]. Positive affect was significantly influenced by Nativeness [*χ*^2^(1) = 20.08, *p* < 0.001], with infants exposed to native stimuli (*M* = 0.078, SD = 0.018) showing higher positive affect infants exposed to non-native stimuli (*M* = 0.059, SD = 0.048). No other effects were significant (Figure 2). These results indicate that facial affect does not differ as a function of differences in behavioral responses indicating discrimination.

#### 3.2.3 Relationship with discrimination scores

**Figure 3:**
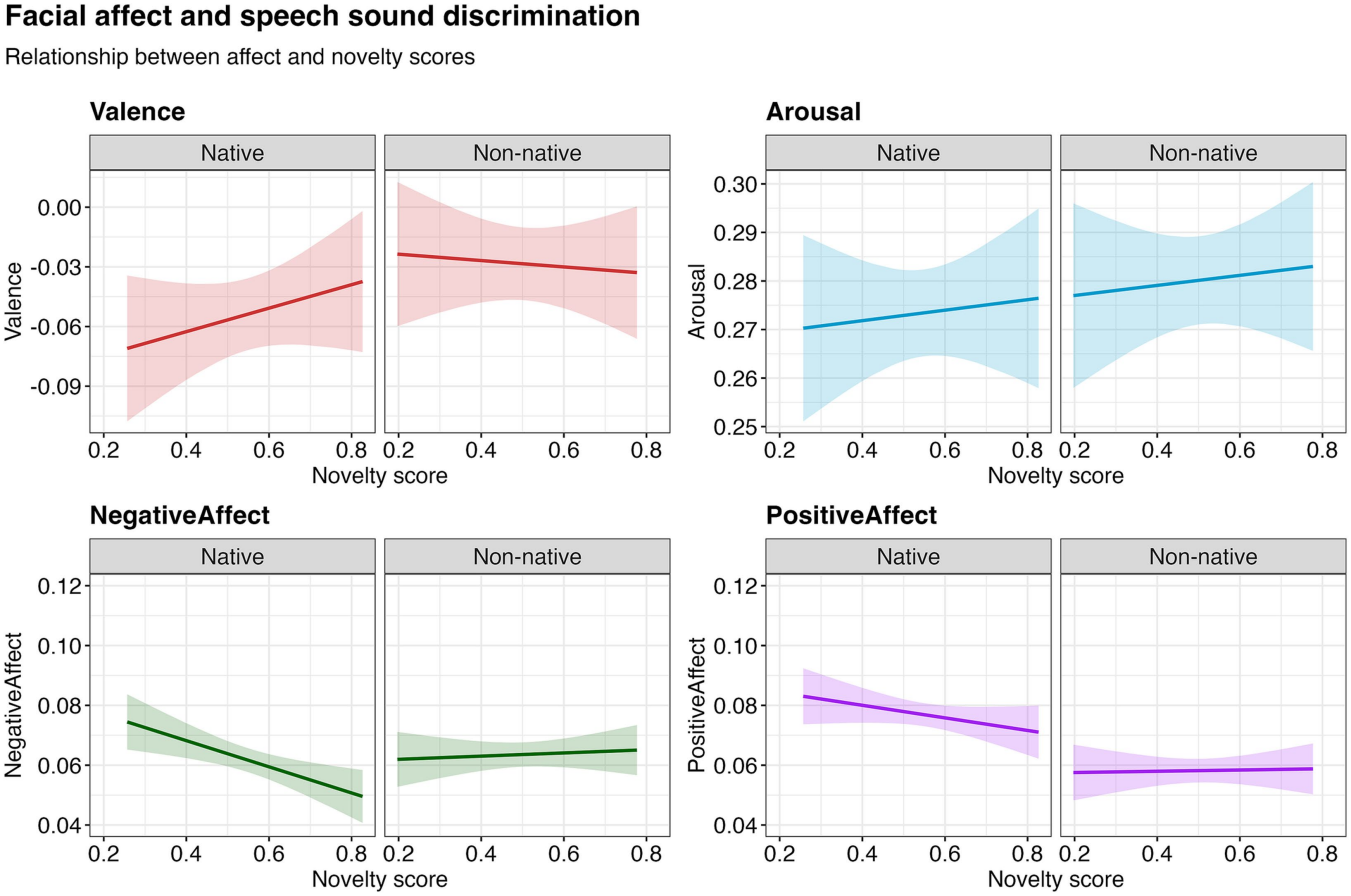
Relationship between facial affect in the habituation phase and novelty score (indicator of speech sound discrimination) in the test phase, based on model estimates. The shaded region represents the 95% confidence interval around the fitted values.

When predicting facial expressions during habituation using gaze-based discrimination scores from the test phase, no significant relationships for valence [*χ*^2^(1) = 0.31, *p* = 0.580], arousal [*χ*^2^(1) = 0.26, *p* = 0.608] or positive affect [*χ*^2^(1) = 0.86, *p* = 0.354] were observed. There was an interaction between the novelty score and nativeness for negative affect [*χ*^2^(1) = 6.15, *p* = 0.0130], and post-hoc analyses revealed that negative affect decreased with higher novelty scores for native speech sounds [*χ*^2^(1) = 9.89, *p* = 0.002], but not for non-native speech sounds [*χ*^2^(1) = 0.19, *p* = 0.665] (Figure 3). These preliminary findings suggest a potential link between facial expressions and language measurement performance.

## 4 Discussion

This study explored infant facial affect during a visual habituation paradigm to examine perceptual attunement to native and non-native speech sounds. By adding facial expression analysis to traditional gaze-based measures, we sought to assess the potential of integrating this measure into the study of perceptual attunement.

Effects of facial affect and arousal were strongest during the habituation phase. Arousal decreased over habituation trials, which is consistent with observations of declining engagement as habituation advances (Hunter and Ames, 1988; Thompson, 2009). Positive affect increased, possibly reflecting a growing preference for familiarized items (Hunter and Ames, 1988). At the same time, global valence as estimated by Baby FaceReader decreased. While we calculated positive affect based on AU12 (lip corner puller), which closely aligns with manual codings of infant positive affect (Zaharieva et al., 2024), the valence score is a composite of several action units that correlates more weakly with manual coding. Future research is needed to establish the relationship of these scores to infant affective expressions.

No significant differences in facial expressions emerged between same and switch trials in the test phase, suggesting that these conditions might not elicit sufficiently strong changes in affective responses.

In our exploratory analysis relating facial expressions to discrimination results, we observed that better discrimination of native contrasts was linked to lower negative affect. This pattern may indicate that successful processing of familiar contrasts leads to reduced frustration or negative responses. Preference for native sounds has also been suggested to reflect expectation of information (Begus et al., 2016), and the present results could indicate that infants who are better in detecting relevant information show better perceptual attunement.

Stimulus nativeness affected facial expressions, such that arousal and valence were higher for non-native than native stimuli in the habituation phase, while positive affect was higher for native stimuli in both habituation and test. Higher overall arousal for non-native stimuli might reflect infants’ weaker familiarity with those stimuli (Hunter and Ames, 1988). The higher positive affect for native stimuli echos the broader literature showing infants’ preferences for native speech sounds (e.g., Jusczyk et al., 1993), but could also be linked to overall higher affective valence of the native stimulus recordings (see stimuli section). The difference in direction of valence and positive affect effects for non-native stimuli reiterates the difficulty of interpreting these. Overall, the differences observed between native and non-native contrasts in the present study are inconclusive, inviting caution in the interpretation of results.

Age-related differences in affective responses were also apparent. For instance, positive affect during habituation was higher for 9-month-old than 12-month-old infants in native, but not non-native trials. These findings might suggest an interplay between developmental changes in perceptual attunement and affective responses.

Overall, this study demonstrates that facial expression analysis can complement traditional measures in studies of perceptual attunement, particularly by capturing affective changes during the habituation phase of laboratory experiments.

This study highlights the potential of facial expression analysis as a tool for tracking the attunement process in laboratory settings. While the learning phase of such studies is typically not the focus of analysis, monitoring facial affect may provide valuable insights into infants’ engagement with speech stimuli and the mechanisms that shape phonetic learning. For instance, in distributional learning studies, infants are exposed to unimodal or bimodal speech sound distributions over a short period, and their ability to categorize speech sounds based on exposure is then assessed (Maye et al., 2002). It has been shown that distributional learning, while still possible outside of sensitive periods, occurs more efficiently within them, requiring less exposure to reach the same learning outcome. Tracking facial expressions during exposure – both inside and outside critical period windows – could provide complementary evidence on the role of affect and arousal in phonetic acquisition during sensitive periods. Similarly, monitoring facial expressions in studies comparing speech sound acquisition from socially enriched versus less enriched stimuli (e.g., Kuhl et al., 2003) could offer new insights into the role of affect and arousal on learning.

Our study also highlighting limitations: not all stimuli elicit detectable differences in facial affect, partly due to the low valence and arousal levels associated with the non-social, non-emotional stimuli in our paradigm. Indeed, the range of valence values in our data largely falls within the neutral/negative range rather than positive (Zaharieva et al., 2024). These constraints likely contribute to the lack of differences in facial expressions during the test phase of our study, must be carefully considered when applying facial affect analysis to studies that are not designed to capture arousal and affect in the first place.

Our findings provide preliminary evidence that facial affect reflects engagement and processing differences during habituation to native and non-native speech sounds. While gaze-based measures remain central to studying perceptual attunement, facial expressions offer a promising, though context-dependent, tool for capturing affective and cognitive dimensions. The data used in the present study are part of a larger data collection including measurements of infants’ socio-communicative environment as well as language outcomes. Relating such measures to facial affect data could deepen our understanding of how infants’ affective responses and engagement during speech sound processing are linked to language learning, and may contribute to the ongoing debate on the extent to which speech sound acquisition is constrained by neural plasticity and whether learning mechanisms differ across developmental windows (Werker and Hensch, 2015). This may provide a new avenue for exploring the connections between environmental influences, individual differences, and language development outcomes.

## Data Availability

The anonymized derived data supporting the conclusions of this article, that is, the BabyFaceReader output files and output files from manual coding of looking behavior, will be made available by the authors whenever caregivers have agreed to data sharing in their consent form. Further enquiries should be directed to the corresponding author(s).
